# Sarcopenia in male patients with head and neck cancer receiving chemoradiotherapy: a longitudinal pilot study

**DOI:** 10.7717/peerj.8617

**Published:** 2020-02-26

**Authors:** Namrata S. Chauhan, Stephen Rajan Samuel, Niranjan Meenar, PU Prakash Saxena, Justin W.L. Keogh

**Affiliations:** 1 Department of Physiotherapy, Kasturba Medical College, Mangalore, Manipal Academy of Higher Education, Manipal, Mangalore, Karnataka, India; 2 Department of Radiation Oncology, Kasturba Medical College, Mangalore, Manipal Academy of Higher Education, Manipal, Mangalore, Karnataka, India; 3 Faculty of Health Sciences and Medicine, Bond University, Robina, QLD, Australia; 4 Human Potential Centre, AUT University, Auckland, New Zealand; 5 Cluster for Health Improvement, Faculty of Science, Health, Education and Engineering, University of the Sunshine Coast, Sunshine Coast, Australia

**Keywords:** Cancer, Chemo-radiation therapy, Deconditioning, Muscle strength, Physical performance, SARC-F, Skeletal muscle mass, Resistance training

## Abstract

**Introduction:**

Muscle wasting conditions such as sarcopenia may be highly prevalent in advanced head and neck cancer (HNC) patients (16–71%), with these prevalence rates substantially greater in those who have received chemo-radiotherapy (CRT). According to the updated European Working Group on Sarcopenia in Older People consensus statement, sarcopenia is defined as the age-related loss of muscle strength, muscle mass and physical performance. The high prevalence of sarcopenia in HNC patients is concerning as it has been associated with substantially increased risk of CRT toxicity, respiratory complications and early mortality. With the high prevalence of HNC and sarcopenia in India and the strong link between sarcopenia and poor HNC patient outcomes, it is important to screen for the presence of sarcopenia in Indian patients receiving CRT for HNC.

**Methods:**

This longitudinal pilot study aimed to routinely monitor 19 men receiving CRT for their HNC for a variety of sarcopenic-related outcomes over three time points during their 7 weeks of CRT. Participants were required to be male, with a minimum age of 30 years, with a Stage III, IVa or IVb diagnosis of HNC and be currently undergoing a 7 weeks course of CRT in an oncology department. Outcomes included probable sarcopenic diagnosis were estimated by the SARC-F, handgrip strength, skeletal muscle mass was estimated by bioelectrical impedance and physical performance was assessed by the Timed Up and Go. Repeated measures ANOVA and Bonferroni post-hoc tests were used to identify significant differences at the three time points with a *p* < 0.05.

**Results:**

The 19 participants in this trial at a mean age of 56.5 ± 10.2 years (range = 39–75 years), with most (*n* = 13, 68.4%) employed in laboring occupations. At baseline, 31.5% (*n* = 6) of the participants already had probable sarcopenia based on their total SARC-F score, with this increasing to 89.4% (*n* = 17) at the end of 7 weeks CRT. In addition, significant decreases in strength, skeletal muscle mass and Timed Up and Go performance were observed, with these declines significantly greater at 7 weeks than 3 weeks after commencing CRT.

**Conclusions:**

Patients with HNC undergoing 7 weeks of CRT showed clinically significant increases in the incidence of probable sarcopenia based on their total SARC-F score as well as clinically significant declines in handgrip strength, skeletal muscle mass and Timed Up and Go performance. Due to the relationship between sarcopenia and a host of adverse events related to CRT in HNC patients, these results suggest that oncologists and their allied health teams should routinely monitor these patients during CRT and provide the relevant exercise therapy and nutritional support to those patients in need.

## Introduction

Current statistics indicate that India accounts for over one sixth of the world’s population. Head and neck cancers (HNC) are one of the most common cancers worldwide and account for the majority (25–30%) of all cancers in India, with rates even higher in Indian men than women (Dandekar et al., 2017). Poor oral hygiene, smoking, smokeless tobacco products, alcohol use and human papilloma virus infections are believed to be some of the major contributors to the high incidence rates for HNC in India (Jemal et al., 2011).

In Indian patients with HNC, the severity of the cancer is typically described using a four stage approach. A single modality treatment (either surgery or radiotherapy) is used in stage I and II (early stage), while multimodality treatment (surgery with radiotherapy (RT) and/or chemotherapy, chemo-radiotherapy (CRT)) is more commonly used in advanced stages (III and IV) (Dandekar et al., 2017). While loco-regional control and good disease specific survival rates are being achieved by CRT, these treatments are associated with unique short-term and long-term adverse events (Pulte & Brenner, 2010). A number of HNC patient groups receiving CRT may also experience substantial declines in muscle mass, muscle strength and physical performance (Ganju et al., 2019; Harada et al., 2016; Ida et al., 2015; Sato et al., 2018), all of which are components of the diagnosis of the geriatric condition, sarcopenia. Of further concern, HNC patients undergoing CRT who develop sarcopenia experience approximately twice the rate of CRT toxicity, six times the rate of respiratory complications and a halving of the progression-free and overall survival rates compared to their peers without sarcopenia (Ganju et al., 2019; Harada et al., 2016; Ida et al., 2015; Sato et al., 2018).

While a number of definitions and diagnostic criteria have been developed for sarcopenia, the European Working Group on Sarcopenia in Older People (EWGSOP) appears to be the most commonly used (Cruz-Jentoft et al., 2010, 2014). Recently, a revised EWGSOP definition and diagnostic criteria (referred to as EWGSOP2) has been developed based on the research conducted since the initial consensus (Cruz-Jentoft et al., 2019). The EWGSOP2 still focuses on the same three sarcopenic outcomes (muscle mass, muscle strength and physical performance), but now identifies muscle strength as a key determinant of sarcopenia and recommends it be assessed first. The EWGSOP2 acknowledges that the assessment of muscle mass by dual energy X-ray absorptiometry, magnetic resonance imaging or bioelectrical impedance ca be expensive and time-consuming for routine clinical use and as a result now recommends the SARC-F as a screening tool for sarcopenia (Cruz-Jentoft et al., 2019), especially in clinical practice.

Internationally, the prevalence of sarcopenia in HNC patients has been demonstrated to be high, although there is considerable between-study variation (16–71%) (Elliott et al., 2017; Ganju et al., 2019; Harada et al., 2016; Ida et al., 2015; Sato et al., 2018). Regardless of these between-study variations, the prevalence of sarcopenia in HNC patient groups appear substantially greater than that observed for community dwelling older adults, for which a prevalence of 1–29% has been reported in 15 studies across nine countries (Cruz-Jentoft et al., 2014).

Of additional concern to Indian HNC cancer patients, Indians have a reduced body mass index, higher percentage body fat and reduced skeletal muscle mass and strength than many other ethnic groups including Caucasians (Kapoor et al., 2019; Kryst et al., 2019; Marwaha et al., 2014). Specifically, an international study involving 18,363 older adults (aged 65 years and older) from three European, three Asian, two African and one South American country, demonstrated higher sarcopenia prevalence rates in older Indians (17.5%) than the other eight countries assessed (12.6–16.7%) (Tyrovolas et al., 2016). A similar study involving 10,892 adults aged 65 years and older from three Asian, two African and one North American country demonstrated that India had the second worst handgrip strength scores, with 50% of older Indian men having below normal grip strength (Brennan-Olsen et al., 2019).

It is therefore possible that as a function of India’s greater sarcopenia prevalence rates and the relative lack of allied health services in Indian hospitals, that Indian HNC patients may be at even greater risk of developing sarcopenia and sarcopenia-related adverse events than those in other more developed countries. Such issues suggest that sarcopenia screening programs may need to considered for at-risk Indian populations such as advanced HNC patients, especially those considering undergoing CRT. One potentially feasible approach may be the routine use of the EWGSOP2-recommended SARC-F (Cruz-Jentoft et al., 2019).

The SARC-F is a self-report tool consisting of five questions (items) that takes ~2 min to complete, with the items focusing on the older individual’s perception of their muscular strength, ability to walk, rise from a chair and climb stairs as well as fall status (Malmstrom & Morley, 2013). Of particular relevance to the study involving Indian HNC patients, the SARC-F has been demonstrated to exhibit reasonable validity and/or reliability when used by older adults across a variety of countries and languages (Bahat et al., 2018; Cao et al., 2014; Ida et al., 2017; Malmstrom et al., 2016; Rodriguez-Rejon et al., 2018).

Due to the size of the Indian population, its high HNC cancer and sarcopenia prevalence and its developing healthcare system, we feel that substantially greater research needs to be conducted on Indian cancer patients to see if the considerable international research can be directly applied to the Indian context. Such research is crucial, as a relatively recent systematic review identified only 13 studies that examined the benefits of exercise for Indian cancer patients (Samuel et al., 2015).

Therefore, the two specific aims of this pilot study were to: (1) quantify sarcopenic-related outcomes in an at-risk population (HNC patients about to undergo CRT); and (2) to gain some insight into how these outcomes may change over 7 weeks of CRT. It was hypothesized that these patients would have relatively normal sarcopenic outcomes when commencing CRT, but that 7 weeks of CRT would result in a substantially greater incidence of probable sarcopenia as identified by the SARC-F and significant declines in muscle mass, muscle strength and physical performance would be observed during CRT.

## Materials and Methods

### Participants

This short-term, longitudinal cohort study sought to quantify the potential change in probable sarcopenia diagnosis (as assessed by the total SARC-F score), muscle mass, strength and physical performance in HNC patients receiving CRT using assessments that are endorsed by EWGSOP2 as valid and feasible for use in clinical practice (Cruz-Jentoft et al., 2019). To be eligible to participate in this study, participants had to at least 30 years old, with a head or neck carcinoma (Stage III, IVa or IVb) and admitted to an oncology department for a 7 weeks course of CRT (radiation of 70 Gy and Cisplatin dose of chemotherapy 40 mg/m^2^), administered on a weekly basis. Participants with orthopedic and neurological problems or an inability to comply with the study procedure were ineligible. Participants who were included in the study were all admitted to the university hospital from December 2018 to February 2019 and screened for inclusion and exclusion after signing the informed consent form. Ethical approval was obtained from the Institutional Research Committee and Ethical Committee from Kasturba Medical College, Mangalore.

### Procedures

In order to ensure consistency of testing time and hydration status, all assessments were conducted on the patients within 1–2 h of finishing their lunch. Participants completed the SARC-F self-report sarcopenia screening tool which consists of five questions, each scored from 0 to 2 (Malmstrom & Morley, 2013). The first four questions assessed walking speed, walking assistance, chair rise and stair climbing ability, with each question graded as: no (0), some (1) or an inability (2) to perform a given task, respectively. The last question assessed falls history in the previous year and was graded as: no falls (0), 1–3 falls (1) and 4 or more falls (2). Total SARC-F scores ≥4 are indicative of probable sarcopenia (Malmstrom et al., 2016).

Skeletal muscle mass was assessed by the OMRON Karada Scan Hbf 375 Body Composition Monitor (Gibson, Heyward & Mermier, 2000). Participants were asked to step onto the measurement platform, by placing the feet on the foot electrodes and to grip the hand electrodes and extend their arms straight at a 90° angle to the body. Muscle strength was assessed by Jamar handgrip dynamometer, with the participants seated, adduction in their shoulder joint, elbow flexed at 90° and forearm in the neutral position. The participant was asked to squeeze the hand dynamometer with their preferred hand as hard as possible for 4–5 s, during 3 trials, each trial separated by one minute (Figueiredo et al., 2007). Physical performance was assessed by the Timed-Up and Go test, wherein the participants were asked to stand up from a stable chair with armrest, walk to and turn around a cone positioned 3 m in front of the chair and regain a seated position as quickly as possible. In this test, participants were allowed to use the armrest if needed during the sit-stand phase. The largest handgrip score and the fastest Timed-Up and Go trial were recorded for data analysis (Shumway-Cook, Brauer & Woollacott, 2000).

### Statistical analysis

A comparison of means of the outcome variables to their standard deviations, indicated that the means were almost always substantially greater and twice the standard deviation, which implies that data was normally distributed (Peat & Barton, 2005). Therefore, descriptive statistics including means and standard deviations were used to describe the participants demographic characteristics and sarcopenic outcomes over the three timepoints (baseline, third week and seventh week of CRT). Repeated measure analysis of variance was performed to identify if any significant differences occurred for any of the dependent variables over the three time points. Wherever significant differences were observed, post–hoc Bonferroni tests were used to identify which timepoints were significantly different. For all statistical tests, a *p*-value ≤ 0.05 was considered to indicate statistical significance.

## Results

The demographics of the 19 participants at recruitment was 56.5 ± 10.2 years, with the majority (*n* = 13, 68.4%) engaged in laboring occupations (see Table 1). At baseline, 31.5% (*n* = 6) of the participants already had probable sarcopenia based on their total SARC-F score, with this increasing to 89.4% (*n* = 17) at the conclusion of seven weeks of CRT (see Table 2). A significant decline in all of the sarcopenic outcomes was observed after three weeks of CRT, with this even more pronounced after seven weeks of CRT.

**Table 1:** Baseline characteristics of the head and neck cancer participants.

Outcome	Value
Age (year)	56.5 ± 0.2
Height (cm)	162.4 ± 7.4
Body mass (kg)	47.2 ± 10.3
Body mass index (kg·m^−2^)	17.8 ± 2.9
Occupation	
Labourer	13 (68.4%)
Agriculturalist	3 (15.8%)
Mechanic	1 (5.3%)
Priest	1 (5.3%)
Tailor	1 (5.3%)
Cancer stage	
Stage III	12 (63.2%)
Stage IV	7 (36.8%)
Cancer location	
Tongue	5 (26.3%)
Hypophyarynx	3 (15.8%)
Buccal mucossa	2 (10.5%)
Oral cavity	2 (10.5%)
Oropharynx	2 (10.5%)
Supraglottis	2 (10.5%)
Tonsil	2 (10.5%)
Tonsillar fossa	1 (5.3%)

**Note:**

All data are presented as means ± standard deviation, with the exception of cancer stage and cancer location, whereby the data is presented as number (percentage).

**Table 2:** Changes in sarcopenic-related outcomes in head and neck cancer participants.

Tools	Baseline	3rd week	7th week
SARC-F	2.6 ± 1.6	3.6 ± 1.5^a^	4.7 ± 1.4^a^^,^^b^
Individuals with probable sarcopenia (i.e. Total SARC-F score ≥ 4.0)	6 (31.5%)	10 (52.6%)	17 (89.4%)
Handgrip strength (kg)	26.1 ± 6.0	23.3 ± 6.2^a^	20.4 ± 5.6^a^^,^^b^
Skeletal muscle mass (kg)	29.9 ± 3.0	28.7 ± 3.3^a^	26.4 ± 3.8^a^^,^^b^
Timed Up and Go (s)	10.1 ± 2.7	10.9 ± 2.9^a^	12.1 ± 3.0^a^^,^^b^

**Notes:**

All data are presented as means ± standard deviation, with the exception of individuals with probable sarcopenia, whereby the data is presented as number (percentage) of participants with probable sarcopenia.

a Significantly different (*p* < 0.05) than baseline.

b Significantly different (*p* < 0.05) than third week.

## Discussion

The major findings of this pilot study were that sarcopenia and its associated adverse events may be highly prevalent in advanced stage (Stage III, IVa or IVb) Indian HNC patients, especially after completing seven weeks of CRT. The tendency for poor sarcopenic outcomes to be observed even prior to starting CRT was quite concerning due to the relatively young (mean age of 56.5 years, range 39–75 years) of the HNC patients in the study. Specifically, prior to starting CRT, close to one third (31.5%) of the participants had probable sarcopenia based on their total SARC-F score ≥ 4 (Malmstrom & Morley, 2013). The sarcopenia prevalence of 31.5% prior to starting CRT was considerably greater than that observed in three studies of community dwelling Indians with mean ages of 70–74 years (8–17%) (Anand et al., 2019; Mohanty & Sahoo, 2016; Tyrovolas et al., 2016). It was also some concern that prior to undergoing CRT, that the mean handgrip strength (26.1 kg) of our participants was fractionally under the below normal (sarcopenic) threshold of 27 kg for men (Cruz-Jentoft et al., 2019). This low level of handgrip strength was consistent with a large international study of 10,892 older participants, in which 50% of Indian men over 65 years of age had below normal handgrip strength (Brennan-Olsen et al., 2019). Collectively, these results indicate that the advanced Indian HNC patients with a mean age of 55 years who were involved in the present study, had a greater prevalence of sarcopenia and comparably poor levels of handgrip strength to that observed in larger studies of Indian older adults aged 65 years and above.

Of even greater concern than the baseline scores, was the significant increase in probable sarcopenia and declines in muscle strength, muscle mass and physical performance across the seven weeks of CRT. For example, the number of HNC patients with probable sarcopenia based on a total SARC-F score ≥4, increased from 31.5% at baseline to 52.6% and 89.4%, after three weeks and seven weeks, respectively of CRT. The rates of sarcopenia for our relatively young participants at seven weeks CRT (89.4%) were approximately five times higher than normative data for India adults over the age of 65 years of 17% (Tyrovolas et al., 2016) and over twice as high as that observed in older Indians (mean age of 70 years) with vertebral fractures (38%) (Anandi et al., 2016). These sarcopenia results also appear substantially greater than the international studies of HNC patients that have indicated sarcopenia prevalence rates of 16–71% (Elliott et al., 2017; Ganju et al., 2019; Harada et al., 2016; Ida et al., 2015; Sato et al., 2018).

The HNC patients also experienced significant declines of ~5.7 kg (22%) in handgrip strength, 3.3 kg (11%) in muscle mass and 2.0 s (20%) in Timed-Up and Go test performance after seven weeks of CRT. Such declines appear of clinical significance as they exceed the minimal clinically important difference (MCID) of 5 kg for handgrip strength (Lang et al., 2008) and ~1.3 s for the Timed-Up and Go test (Alghadir, Anwer & Brismée, 2015; Wright et al., 2011). Collectively, the increased total SARC-F scores and declines in handgrip strength, muscle mass and Timed-Up and Go test performance are reflective of increased impairment and disability in this patient group that have the potential to reduce the patients’ general well-being and overall quality of life. Beyond these functional consequences of these losses in muscle strength, muscle mass and physical performance, such declines have also been associated with significantly greater risk of CRT toxicity, respiratory complications and declines in progression-free and overall survival in HNC patients (Ganju et al., 2019; Harada et al., 2016; Ida et al., 2015; Sato et al., 2018).

As a result of these clinically significant declines in muscle strength, muscle mass and physical performance observed during seven weeks of CRT and the association between sarcopenia and a host of adverse events in HNC patients, oncologists and their allied health care teams, especially those in India, may need to consider a number of alterations to the usual care strategies for these patients. The first may be the implementation of routine monitoring of some of the sarcopenic-related outcomes used in the present study to help identify patients who are at risk of sarcopenia and the associated adverse events (Baxi, Schwitzer & Jones, 2016; Ganju et al., 2019). Such monitoring may be useful to implement on at least an annual basis with advanced HNC patients, especially those considering CRT, as sarcopenia can reduce CRT compliance, and increase their risk of adverse events and early mortality. Based on simplicity and clinical relevance of the recommendations of the EWGSOP2, we would recommend that the oncology healthcare teams should routinely assess SARC-F and/or handgrip strength test (Cruz-Jentoft et al., 2019), with such assessments only taking a handful of minutes to complete. It is even possible that the SARC-F monitoring could be performed by the patient themselves, as a recent study demonstrated that changes in older adults’ SARC-F scores match that of quantitative assessments of muscle strength and physical performance performed by their allied health professionals (Keogh et al., 2019).

For those HNC patients who are diagnosed with sarcopenia and appear at risk of developing sarcopenic adverse events, clinicians should look to ensure their patients have exercise rehabilitation and/or nutritional support. Specifically, clinicians should look to follow the recommendations of a Society of Sarcopenia, Cachexia and Wasting Disorders position statement, by ensuring these patients complete 2–3 sessions per week of progressive resistance training and obtain protein intakes of up to 1.5 g/kg bodyweight per day to offset these sarcopenic-related treatment effects (Bauer et al., 2019). Consistent with these guidelines, exercise programs involving substantial components of progressive resistance training have been demonstrated to be safe and feasible, and able to significantly increase muscle strength, physical performance and quality of life and approach statistical significance for reduced fatigue in international (Grote et al., 2018) and Indian (Samuel et al., 2019, 2013) studies of HNC patients. Such results further support the importance of physiotherapists providing exercise therapy to Indian HNC patients undergoing CRT, with dietitians also having an important role to ensure these patients obtain sufficient protein (Bauer et al., 2019).

## Conclusions

The results of this pilot study demonstrated that even prior to receiving CRT, Indian Stage III, IVa or IVb HNC patients may be at elevated risk of sarcopenia (compared to their age-matched peers) and in developing sarcopenic-related adverse events, with this becoming much more pronounced at the completion of seven weeks of CRT. As this is only a pilot study involving a small number of male patients, it would be recommended that future studies are conducted in order to confirm the exploratory results presented in this manuscript. If similar findings are observed in future studies, it is recommended that oncologists work closely with physiotherapists and dieticians to routinely monitor these patient groups for sarcopenia and intervene with evidence-based exercise and nutritional support where required.

## Supplemental Information

10.7717/peerj.8617/supp-1 Supplemental Information 1 Raw data.

## Additional Information and Declarations

### Data Availability

The following information was supplied regarding data availability: The data that was used to perform the descriptive and inferential statistic is available as a Supplemental File.
